# Stable Toll-Like Receptor 10 Knockdown in THP-1 Cells Reduces TLR-Ligand-Induced Proinflammatory Cytokine Expression

**DOI:** 10.3390/ijms17060859

**Published:** 2016-06-01

**Authors:** Hai Van Le, Jae Young Kim

**Affiliations:** Department of Life Science, Gachon University, Seongnam, Kyeonggi-Do 461-701, Korea; levan.hus@gmail.com

**Keywords:** toll-like receptor 10, knockdown, THP-1, pro-inflammatory cytokines

## Abstract

Toll-like receptor 10 (TLR10) is the only orphan receptor whose natural ligand and function are unknown among the 10 human TLRs. In this study, to test whether TLR10 recognizes some known TLR ligands, we established a stable TLR10 knockdown human monocytic cell line THP-1 using *TLR10* short hairpin RNA lentiviral particle and puromycin selection. Among 60 TLR10 knockdown clones that were derived from each single transduced cell, six clones were randomly selected, and then one of those clones, named E7, was chosen for the functional study. E7 exhibited approximately 50% inhibition of *TLR10* mRNA and protein expression. Of all the TLRs, only the expression of TLR10 changed significantly in this cell line. Additionally, phorbol 12-myristate 13-acetate-induced macrophage differentiation of TLR10 knockdown cells was not affected in the knockdown cells. When exposed to TLR ligands, such as synthetic diacylated lipoprotein (FSL-1), lipopolysaccharide (LPS), and flagellin, significant induction of proinflammatory cytokine gene expression including *Interleukin-8* (*IL-8*), *Interleukin-1 beta* (*IL-1β*), *Tumor necrosis factor-alpha* (*TNF-α*) and *Chemokine* (*C–C Motif*) *Ligand 20* (*CCL20*) expression, was found in the control THP-1 cells, whereas the TLR10 knockdown cells exhibited a significant reduction in the expression of *IL-8*, *IL-1β*, and *CCL20*. TNF-α was the only cytokine for which the expression did not decrease in the TLR10 knockdown cells from that measured in the control cells. Analysis of putative binding sites for transcription factors using a binding-site-prediction program revealed that the TNF-α promoter does not have putative binding sites for AP-1 or c-Jun, comprising a major transcription factor along with NF-κB for TLR signaling. Our results suggest that TLR10 is involved in the recognition of FSL-1, LPS, and flagellin and TLR-ligand-induced expression of *TNF-α* does not depend on TLR10.

## 1. Introduction

Toll-like receptors (TLRs) are innate immune receptors responsible for detecting host invasion by pathogens. Among the 10 TLRs discovered in humans, TLR10 is the only orphan receptor whose natural ligand and function are unknown. One of the reasons for this gap in our knowledge is that the mouse *TLR10* gene is a non-functional pseudogene [1] and thus knockout technology, which can be used for a loss-of-function study, is not applicable for TLR10. Therefore, a viable alternative would be the use of human TLR10 knockdown immune cells for functional study. TLR10 has been shown to have approximately 50% amino acid sequence homology with TLR1 and TLR6 [2]. A co-immunoprecipitation study demonstrated that TLR10 forms a heterodimer with a TLR1 or TLR2, suggesting that TLR10 may act as a co-receptor to these TLRs and therefore share the same family of ligands [3]. In addition, a study using computational modeling suggested that a TLR2/10 dimer recognizes triacylated lipopeptides, while a TLR1/10 dimer or a TLR10 homodimer senses diacylated lipopeptides [4]. Recent studies have reported possible natural ligands for TLR10. Studies with human TLR10 knockdown monocytic cell lines have suggested that TLR10 acts as an immune sensor for the influenza virus [5] and *Listeria monocytogenes* [6]. In addition, a study using gastric epithelial cells from patients infected by Helicobacter pylori proposed that TLR10 forms a heterodimer with TLR2 and acts as a functional receptor recognizing *Helicobacter pylori* lipopolysaccharide (LPS) [7]. These previous studies all suggested that TLR10 acts as an immune sensor for pathogens much like other TLRs do.

However, some studies have suggested that TLR10 function may differ from that of other TLRs. The first functional difference is that TLR10 does not appear to induce NF-κB activation, which is a typical TLR signaling process, although it does interact with MyD88, a common intracellular signaling molecule in TLR pathways [1,3,8]. Second, TLR10 exhibits restricted expression in lymphoid tissues [2,9,10] and in regulatory T cells under the control of the transcription factor FoxP3 [11]. The third difference is that blocking of the TLR10 molecule by selective antibody increases the proinflammatory cytokine production of human peripheral mononuclear cells in response to the addition of the TLR2/1 ligand pam3CSK4, and in addition, transgenic mice expressing human TLR10 produce lower amounts of inflammatory cytokines in response to TLR2/1 agonist, suggesting a possible anti-inflammatory role for TLR10 [12].

In this study, in order to investigate the controversial issue of the potential function of TLR10, we established a stable TLR10 knockdown human monocytic cell line and examined the proinflammatory cytokines expression of these cells in response to TLR ligands such as synthetic diacylated lipoprotein (FSL-1), lipopolysaccharide (LPS), and flagellin.

## 2. Results

### 2.1. Toll-Like Receptor 10 (TLR10) Short Hairpin RNA (shRNA) Lentiviral Particle-Mediated TLR10 Knockdown in THP-1 Cells

**Figure 1:**
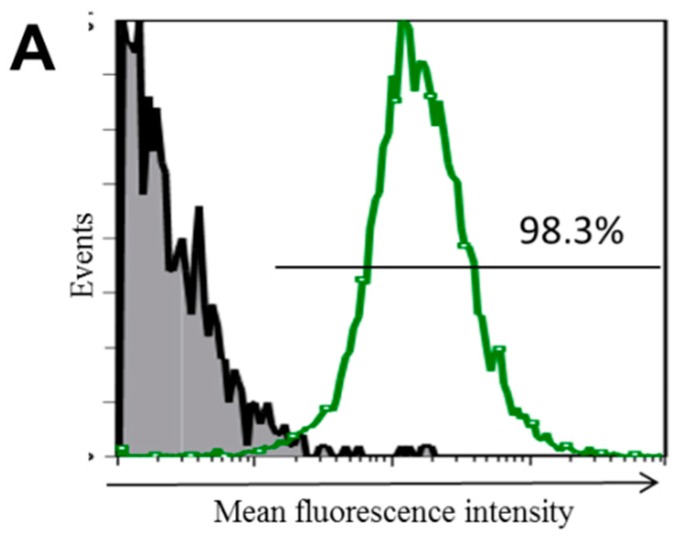

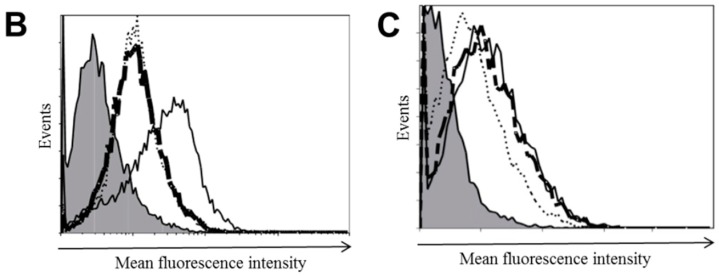
High transduction efficiency of green fluorescent protein (GFP)-expressing lentiviral particles and short hairpin RNA lentiviral particle-mediated silencing of Toll-like receptor 10 (TLR10) in THP-1 cells. (**A**) THP-1 cells were transduced with copGFP control lentiviral particles at a multiplicity of infection of 10:1 and underwent puromycin selection for 20 days. GFP expression was analyzed by fluorescence-activated cell sorting (FACS); Intracellular TLR10 expression (**B**) and extracellular TLR10 expression (**C**) were analyzed by FACS. THP-1 cells were transduced with TLR10 shRNA lentiviral particles with same experimental conditions used in GFP transduction. Gray shaded area, negative control omitting the primary antibody; histograms with thick dashed line and dotted line, two independent sets of TLR10 shRNA transduced cells; histogram with solid line, control shRNA-transduced cells.

To examine the transduction efficiency of the shRNA, THP-1 cells were transduced with the green fluorescent protein (GFP) control lentiviral particles at a multiplicity of infection (MOI) of 10:1. The transduced cells were selected in the presence of puromycin for 20 days and were then analyzed for GFP expression using flow cytometry (Figure 1A). More than 98% of the cells expressed GFP, indicating great transduction efficiency. In order to knockdown TLR10 expression, the THP-1 cells were transduced with *TLR10* shRNA lentiviral particles as described in the Experimental Section. After 20 days of puromycin selection, TLR10 expression was analyzed by fluorescence-activated cell sorting (FACS). The mean fluorescence intensity of the intracellular TLR10 expression in the TLR10 shRNA-transduced cells from two independent cultures were 15.5 and 16.4 (thick dashed and dotted lines in Figure 1B), while that of the control lentiviral particle transduced cells was 41.3 (solid line in Figure 1B), indicating an approximately 60% inhibition of the intracellular TLR10 expression in the shRNA-transduced cells. The extracellular TLR10 expression of the TLR10 shRNA-transduced cells was decreased from that of the control cells by approximately 20% (Figure 1C).

### 2.2. Generation of Stable TLR10 Knockdown THP-1 Clones

**Figure 2:**
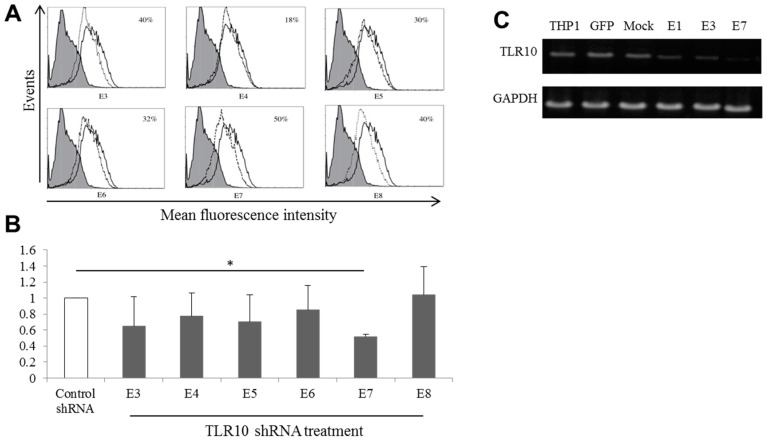
Establishment of stable TLR10 knockdown THP-1 clones using *TLR10* shRNA lentiviral particles. To create stable a knockdown clone from single cell, puromycin-resistant cells were selected, transferred individually into multiwall dishes, and expanded in the presence of puromycin for an additional month. Six clones were randomly chosen from a total of 60 clones and were analyzed for TLR10 expression. (**A**) Intracellular TLR10 expression of the THP-1 cells. Gray shaded area, negative control; histogram with dotted line, *TLR10* shRNA transduced cells; histogram with solid line, control shRNA-transduced cells; (**B**) *TLR10* mRNA expression in TLR10 knockdown clones was analyzed by a quantitative real time-PCR (qRT-PCR). Data are means of the relative expression ratio ± SD. * *p* < 0.001 *vs.* control; (**C**) Representative agarose gel electrophoresis image showing *TLR10* mRNA expression of THP-1 cells analyzed by semi-quantitative PCR. To verify the long-term silencing effects of TLR10 shRNA lentiviral particles, after 8 months of continuous subcultures, the *TLR10* mRNA expression of the E1, E3, and E7 clones was analyzed by semi-quantitative PCR. GFP: copGFP control lentiviral particle-transduced cells; Control shRNA: control shRNA lentiviral particle-transduced cells.

To create stable knockdown clones, puromycin-resistant cells were selected, transferred individually into multiwall dishes, and expanded in the presence of puromycin for an additional month. Six clones were randomly chosen from the 60 clones that were maintained, and these six were analyzed for intracellular TLR10 expression. The mean fluorescence intensities of the six selected clones (dotted lines in Figure 2A) were 27.2, 34.9, 30.6, 30.0, 25.4, and 26.8, while that in the control shRNA-transduced cells (solid line in Figure 2A) was 41.3, indicating approximately 18%–50% inhibition of the intracellular TLR10 expression in the clones. A quantitative real timePCR (qRT-PCR) analysis revealed that the clone E7 was the most efficient knockdown clone among the six tested (Figure 2B). To verify the long-term silencing effects of TLR10 shRNA lentiviral particles, after 8 months of continuous sub-culturing, the *TLR10* mRNA expression of the selected clones was examined and was found to be stably suppressed, especially in the E7 clone (Figure 2C). To determine the effect of TLR10 knockdown on the THP-1 cell proliferation, we examined the growth rate of TLR10 knockdown cells using both trypan blue exclusion and Ez-Cytox assay. As shown in Figure S1, there was no difference in the growth rate between control and TLR10 knockdown cells.

### 2.3. Stable Knockdown of TLR10 Does Not Affect the Expression of Other TLRs and Phorbol 12-Myristate 13-Acetate (PMA)-Induced Differentiation of THP-1 Cells

**Figure 3:**
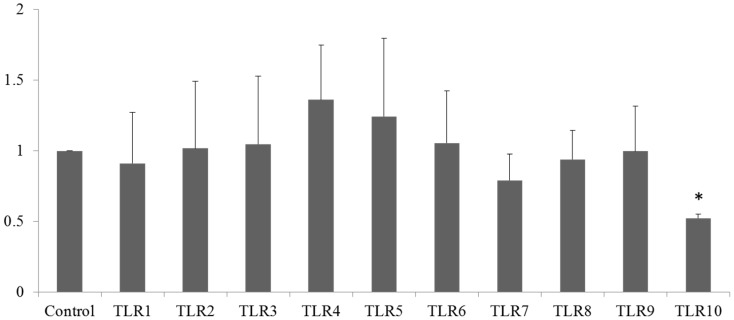
Knockdown of TLR10 does not affect the expression of other TLRs. mRNA expression of *TLR1* to *TLR10* of the E7 TLR10 knockdown clone was determined by quantitative real-time PCR. Control designates cells infected with control shRNA lentiviral particles. Data are means of the relative expression ratio ± SD. * *p* < 0.001 *vs.* control.

To determine whether transduction of the *TLR10* shRNA lentiviral particles to the THP-1 cells had effects on the expression of other TLRs, the expression of the 10 human TLRs in the E7 clone was examined by qRT-PCR. As shown in Figure 3, *TLR10* mRNA expression was significantly suppressed but the expression levels of the other TLRs were not significantly affected, indicating selective suppression of TLR10 expression.

**Figure 4:**
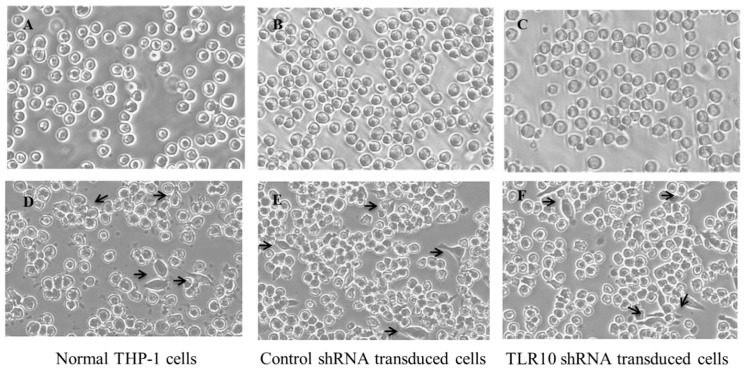
Knockdown of TLR10 does not affect the PMA-induced differentiation of THP-1 cells. One million cells were treated with 50 ng/mL of PMA for 24 h and were then observed by light microscopy (100× magnification). The arrows indicate differentiated cells. (**A**–**C**) PMA untreated; (**D**–**F**) PMA treated.

Since TLRs are critical for the differentiation of human monocytes into macrophages [13], it was necessary to examine whether the TLR10 knockdown affected the differentiation of the THP-1 cells into macrophages. For this, E7 cells were treated with phorbol 12-myristate 13-acetate (PMA) which is routinely used for differentiation of THP-1 monocytes into macrophages for 24 h and were then observed under a light microscope. There was no significant change in the cell morphology among the non-transduced, control shRNA-transduced, and *TLR10* shRNA-transduced cells, indicating that TLR10 knockdown itself did not influence PMA-induced THP-1 differentiation (Figure 4).

### 2.4. Knockdown of TLR10 Reduces TLR Ligand-Induced Pro-Inflammatory Cytokine Expression

**Figure 5:**
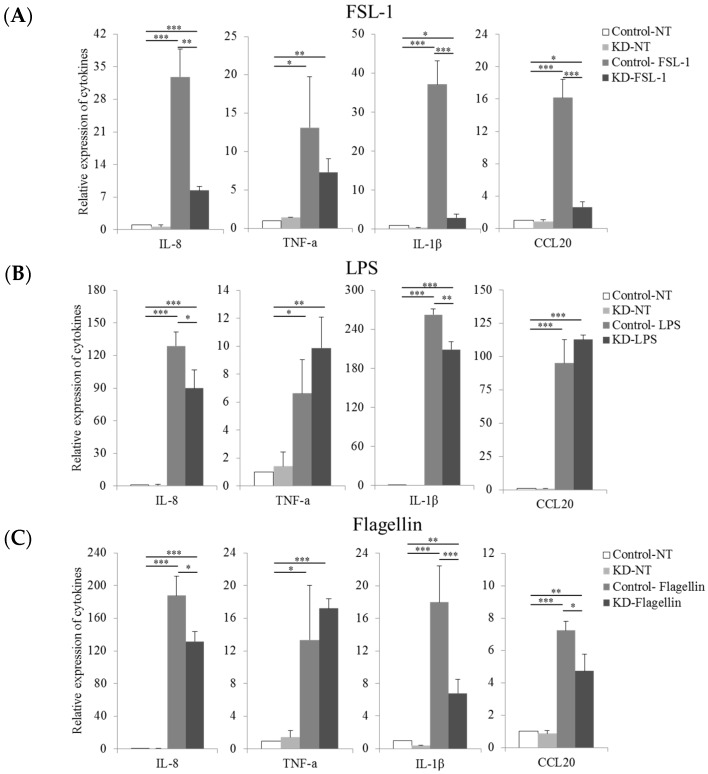
Knockdown of TLR10 reduces TLR-ligand-induced pro-inflammatory cytokine expression. TLR10 knockdown cells were treated with 100 ng/mL of synthetic diacylated lipoprotein (FSL-1), lipopolysaccharide (LPS) or flagellin for 4 h and were then analyzed for *Interleukin-8* (*IL-8*), *Tumor necrosis factor-α* (*TNF-α*), *IL-1β*, and *Chemokine* (*C-C motif*) *ligand 20* (*CCL20*) mRNA expression by real-time quantitative reverse transcription PCR (qRT-PCR). (**A**) FSL-1, (**B**) LPS, and (**C**) flagellin. Control: control shRNA lentiviral particle transduced cells; KD: TLR10 knockdown cells; NT, non-treated. Data are means of the relative expression ratio ± SD. * *p* < 0.05, ** *p* < 0.01, *** *p* < 0.001.

Although recent attempts to discover novel TLR10 ligands in the form of pathogens or pathogenic products have been reported [5,6,7], it still remains to be determined whether TLR10 is capable of recognizing known ligands for other TLRs. Since it is known that cell-surface TLRs form a homo- or heterodimer, whereas endosomal TLRs form a homodimer only [14] and that TLR10 may form a heterodimer with other TLRs as described in the introduction, we wanted to examine a possible involvement of TLR10 in response to ligands for surface TLRs as a co-receptor for other surface TLRs. In our previous study, surface expression of TLR2, TLR4, TLR5 and TLR10 was found in THP-1, while little (TLR6) or no (TLR1) expression of other TLRs was detected [15]. Thus, we tested three different TLR ligands: FSL-1 (TLR2/6 ligand), LPS (TLR4 ligand), and flagellin (TLR5 ligand), as possible ligands for the TLR10 molecule. Since it is known that different TLRs are cooperatively involved in recognition of same pathogenic product, whereas they may negatively regulate each other [16], we treated cells with TLR ligands for only 4 h to avoid or minimize the effects of the possible TLR cross-talk. To confirm whether 4 h-treatment does not induce any change of surface expression of other TLRs, we examined cell surface TLR2, 4, and 5 expression after 4 h-treatment of FSL-1, LPS and flagellin, respectively. As shown in Figures S2 and S3, expression of *TLR2*, *TLR4*, and *TLR5* was not changed at all after stimulation with FSL-1, LPS or flagellin. After treatment with these TLR ligands, the cells were analyzed for the mRNA expression of pro-inflammatory cytokines such as *IL-8*, *IL-1β*, *TNF-α*, and *CCL20* using qRT-PCR. Exposure to FSL-1 caused a marked increase in cytokine expression in the control THP-1 cells; in contrast, the TLR10 knockdown cells exhibited significantly suppressed expression of IL-8, IL-1β, and CCL20 (Figure 5A). However, the expression levels of TNF-α were not significantly different between the FSL-1-exposed control and knockdown THP-1 cells. In response to LPS, the expression levels of IL-8 and IL-1β were found to be reduced in the TLR10 knockdown cells from those levels in the control cells, but no differences in the expression levels of *TNF-α* or *CCL20* were found (Figure 5B). Similar to FSL-1, flagellin exposure led to a significant reduction in the expression of *IL-8*, *IL-1β*, and *CCL20* in the TLR10 knockdown cells from those levels in the control cells (Figure 5C). These results suggest that TLR10 is involved in the recognition of FSL-1, LPS, and flagellin.

### 2.5. Putative Binding Sites of the Nuclear Factor NF-κB, Activator Protein 1 (AP-1) and c-Jun on Promoters of Interleukin-8 (IL-8), Tumor Necrosis Factor-α (TNF-α), IL-1β, and Chemokine (C-C Motif) Ligand 20 (CCL20)

To identify the reason why only the expression of *TNF-α* induced by the TLR ligands tested remained unchanged between the control and TLR10 knockdown cells, we screened for the putative binding sites of transcription factors within the *IL-8*, *IL-1β*, *TNF-α*, and *CCL20* gene promoters using a transcription factor binding-site-prediction program, as described in the experimental section. Since TLRs elicit conserved inflammatory pathways, culminating in the activation of NF-κB and AP-1, and among the AP-1 family proteins, c-Jun is thought to play central roles in inflammatory responses [17,18], we screened for the putative binding sites of NF-κB, AP-1, and c-Jun within each cytokine promoter. As shown in Table 1, putative binding sites for NF-κB were identified within the *IL-8*, *TNF-α*, and *CCL20* proximal promoters (within 250 base pairs up-stream of the start site) but not within the *IL-1β* promoter. Putative binding sites for either AP-1 or c-Jun were identified within the *IL-8*, *IL-1β*, and *CCL20* promoters but not within the *TNF-α* promoter, suggesting that unlike the other cytokines tested, *TNF-α* expression is mainly controlled by NF-κB and is independent of AP-1 [18].

## 3. Discussion

THP-1 cells are widely used as a model system of human monocytes and are known to express TLR10 [6,15,19]. However, genetic manipulation of THP-1 cells through non-viral transfection methods such as liposome- and electroporation-mediated transfection has some limitations, including low transfection efficiency, low cell viability, and the difficulty of stable transfection [20,21]. Therefore, we used lentiviral-particle-expressing shRNA against TLR10 to achieve stable knockdown of TLR10 expression in the current study. Stable knockdown of TLR10 in our cell line was found to be maintained for longer than 8 months, indicating the usefulness of lentiviral-particle-mediated stable knockdown of genes in THP-1 cells over long periods of time. Since the aim of the current study was to determine whether TLR10 is capable of recognizing some known TLR ligands, the selective knockdown of TLR10 without affecting any other TLRs should be guaranteed. In this regard, our TLR10 knockdown cell line, in which the expression of other TLRs and typical PMA-induced macrophage differentiation were not significantly changed after TLR10 knockdown, could be a useful model system to study TLR10 function.

The functional experiments carried out in this study revealed that when TLR10 knockdown cells were challenged with FSL-1, LPS, or flagellin, *IL-8* and *IL-1β* expression was significantly reduced from that in the control cells. These results suggest that TLR10 is involved in the recognition of FSL-1, LPS, and flagellin and in the induction of proinflammatory cytokines, although it is still unknown whether TLR10 is involved in this recognition as a homodimer or heterodimer with other TLRs. Thus, the possible ligand spectrum of TLR10 can be expanded to FSL-1 and flagellin in addition to the previously reported ligand, LPS [7]. However, the result that the expression of *TNF-α*, a primary proinflammatory cytokine, was not altered leads us to speculate that TLR-ligand-induced *TNF-α* expression does not depend on TLR10, and the signaling mechanisms leading to the induction of *TNF-α* may differ from those of *IL-8*, *IL-1β*, and *CCL20*. A previous study suggested that the regulation of *TNF-α* expression by NF-κB in response to LPS exposure in human macrophages is much stronger than the regulation by other transcription factors [18]. Similarly, our result that among the four inflammatory cytokine promoters that were tested, only the *TNF-α* promoter had no putative binding sites for AP-1 or c-Jun led us to suggest that TLR-ligand-induced TLR10 signaling mainly communicates with the AP-1 signaling pathway, although NF-κB is a major transcription factor that is activated downstream of TLR signaling. Suggestions in support of these conclusions have been made in the past as well. For example, it was previously proposed that TLR10 does not appear to induce NF-κB activation, despite its interaction with the common intracellular signaling molecule [1,3,8]. IL-8 [22] and CCL20 [23] are chemokines responsible for the migration of neutrophils, and IL-1β differs from TNF-α in its chemotactic function in macrophages [24], although it shows a striking overlap of biologic activities with TNF-α [25]. Our results showing that the expression of these cytokines possessing chemotactic activity, with the exception of TNF-α, was markedly decreased in TLR10 knockdown cells led us to cautiously suggest that human monocyte TLR10 mainly plays a role in the recruitment of neutrophils and macrophages in response to the recognition of bacterial TLR ligands such as FSL-1, LPS, or flagellin. However, to prove this hypothesis, further experiments utilizing transcription factor reporting assays and cell migration assays after the transfection of the TLR10 gene together with other TLR genes into non-immune cells, such as HEK-293 cells, should be performed.

In summary, in the present study we successfully established a TLR10 knockdown human monocyte cell line using lentiviral particles and found that TLR10 knockdown in this cell line reduced the FSL-1-, LPS-, and flagellin-induced expression of inflammatory cytokines such as *IL-8*, *IL-1β*, and *CCL20*, suggesting that TLR10 is involved in the recognition of these TLR ligands. In addition, we showed that the induction of *TNF-α* expression in response to these TLR ligands does not depend on TLR10 recognition.

## 4. Experimental Section

### 4.1. Reagents and Chemicals

Lipopolysaccharide (LPS) from *Escherichia coli* (*E. coli*) O111:B4 was purchased from Sigma-Aldrich (St. Louis, MO, USA). Synthetic bacterial diacylated lipopeptide, FSL-1 (Pam2CGDPKHPKSF) and flagellin from Salmonella typhimurium were obtained from InvivoGen (San Diego, CA, USA). Antibiotic-Antimycotic, HEPES (4-(2-hydroxyethyl)-1-piperazine ethane sulfonic acid) buffer, and β-mercaptoethanol were purchased from Invitrogen Corp (Gibco BRL, Gaithersburg, MD, USA).

### 4.2. Cell Culture and Diffrentiation

The human monocytic cell line THP-1 was obtained from ATCC (Manassas, VA, USA). THP-1 cells were grown in RPMI-1640 supplemented with 10% heat-inactivated fetal bovine serum, 1% Antibiotic-Antimycotic, 10 mM HEPES buffer, and β-mercaptoethanol at 37 °C in a 5% CO_2_ humidified incubator.

To characterize the differentiation of THP-1 monocytes to macrophages, six-well, flat-bottomed culture plates were filled with 1 × 10^6^ THP-1 cells in 2 mL of culture medium. The cells were incubated in the presence or absence of 50 ng/mL of PMA at 37 °C for 24 h and were then observed under a microscope (TI-SM Nikon, Tokyo, Japan) at 100× magnification.

### 4.3. shRNA-Mediated Gene Silencing

TLR10 knockdown THP-1 cell lines were established using stable expression of short hairpin RNAs (shRNAs) that target *TLR10* mRNA. THP-1 cells were transduced with *TLR10* shRNA lentiviral particles (sc-40272-V), control shRNA lentiviral particles (sc-108080), or copGFP control lentiviral particles (sc-108084) purchased from Santa Cruz Biotechnology, Inc. (Dallas, TX, USA). The sequences of the *TLR10* shRNAs are listed in Table 2. The main target of TLR10 shRNAs is the last exon of *TLR10* mRNA. We initially tested the optimal concentration of the selection agent (puromycin dihydrochloride, sc-108071, Santa Cruz (Dallas, TX, USA)) required to kill 100% THP-1 cells. The cells (2 × 10^5^) were incubated in 3 mL of growth medium with or without puromycin (0.1–10 µg/mL) for 48 h. The selective medium was changed every 2 days. Cell survival was assessed by trypan blue staining every 2 days. After 3 to 5 days of antibiotic selection, most of the cells were found to be dead in the presence of 1 µg/mL of puromycin, so this concentration was used for the selection protocol. Transductions of THP-1 cells were carried out in the presence of 8 µg/mL of polybrene (sc-134220, Santa Cruz (Dallas, TX, USA)). After mixing with polybrene, the viral stocks were added to the cells (1 × 10^4^ cells/well in 96 well plates) at multiplicity of infection (MOI) of 10. After 24 h of transduction, the cells were collected and then fresh media lacking polybrene were added to them. The transduced cells were allowed to proliferate until a sufficient cell number was reached for puromycin selection, which was performed in order to select stable clones expressing the shRNA. The cell culture medium was replaced with fresh medium plus 1 µg/mL of puromycin every 2 to 3 days until resistant clones appeared. After 3–4 weeks, the cells were collected and examined for GFP and TLR10 expression of the cells using FACS analysis. The selected clones were maintained in fresh puromycin-containing medium for an additional month, analyzed, and used for further experiments.

### 4.4. Flow Cytometric Analysis

To investigate the surface expression of TLR10, the cells were incubated with a purified anti-TLR10 antibody (IMG-386A) for 45 min and were then incubated with phycoerythrin-conjugated secondary antibody (goat IgG, CLCC35004, Cedarlane Lab, Burlington, ON, Canada) at 4 °C for 45 min. After washing with phosphate-buffered saline (PBS), the cells were re-suspended in PBS before analysis on a Cytomics FC500 MLP (BeckmanCoulter Inc., Fullerton, CA, USA).

To measure the intracellular TLR expression, an IC-Flow (an intracellular staining flow assay) kit (InvivoGen) was used to fix and permeabilize the cells. One million cells were suspended in 50 µL-fixation buffer and kept at room temperature for 30 min. After a brief spin down, the cells were re-suspended in 50 µL-permeabilization buffer. The cells were stained with primary antibody diluted in permeabilization buffer for 40 min and were then centrifuged, followed by washing twice with permeabilization buffer. The cells were incubated with secondary antibody for 45 min at room temperature, centrifuged, and washed three times with permeabilization buffer. Finally, 0.4 mL of staining buffer was added to the resulting cell pellet and the cell solution was transferred to tubes and cells were analyzed by FACS.

To measure the GFP expression, 1 × 10^6^ cells were collected, washed, re-suspended in PBS, and analyzed by FACS.

### 4.5. Quantitative Real-Time PCR and Semi-Quantitative PCR

In order to analyze the expression of cytokines, such as TNF-α, IL-8, IL-1β and CCL20 in the TLR10 knockdown THP-1 cells in response to TLR ligands, the cells were treated with 100 ng/mL LPS, FSL-1, or flagellin for 4 h and then total mRNA was isolated and analyzed by quantitative real-time PCR (qRT-PCR). The extraction of total RNA was performed using Qiagen RNeasy kit (Qiagen, Valencia, CA, USA). RNA concentrations were determined with a SD2000 micro spectrophotometer (Bioprince, Atlanta, GA, USA). cDNA was synthesized from 2 µg of total RNA with MMLV reverse transcriptase (Bioprince) using an oligo dT primer (Bioprince) at 65 °C for 1 h.

qRT-PCR was performed on the iQ5 multicolor Real-Time PCR detection system (Bio-Rad, Hercules, CA, USA) using the iQ SYBR Green Supermix (Bio-Rad). The sequences of the specific primer set are summarized in Table 3. Sample normalization was performed using the human β-actin gene as an endogenous control. For each sample, the relative abundance of target mRNA was calculated from the Δ*C*_t_ values for the target and endogenous reference gene *β-actin* using the 2-ΔΔ*C*_t_ cycle threshold method.

For semi-quantitative PCR, Taq Polymerase (Beams Biotechnology, Seongnam, Korea) was used. Amplification was carried out using the following primer sets: TLR10, forward 5′-TGGGACGACCTTTTCCTTAT-3′ and reverse 5′-CAGAGATGGGCTGAGAATGAAG-3′ Glyceraldehyde 3-phosphate dehydrogenase (GAPDH), forward 5′-ACAGCCTCAAGATCATCAGCAAT-3′ and reverse 5′-AGGAAATGAGCTTGACAAAGTGG-3′ on a TaKaRa PCR Thermal Cycler (Shiga, Japan). The PCR products were electrophoresed on 1.5% agarose in TBE buffer and visualized using a UV trans-illuminator (Seongnam, Korea).

### 4.6. Prediction of Transcription Factor Binding Sites

To identify putative binding sites for NF-κB, AP-1, and c-Jun within the *IL-8*, *IL-1β*, *TNF-α*, and *CCL20* promoter region, the transcription factor binding site prediction program [26] Alggen Promo software, V3.0.2 [27] was used with the default setting of 15% dissimilarity value.

### 4.7. Statistical Analysis

Data were analyzed by one-way analysis of variance (ANOVA) followed by post hoc comparisons with either the Tukey HSD (honestly significant difference test) for groups of data with equal variances or, alternatively, with the Games-Howell test in one-way ANOVA analyses with unequal variances using SPSS 12.0 for Windows (Armonk, NY, USA). Values are expressed as the means ± standard deviation (SD). Statistical significance was defined as a *p* < 0.05.

## Figures and Tables

**Table 1:** Putative binding sites for the nuclear factor NF-κB, NF-κB1, activator protein 1 (AP-1) and c-Jun in promoters of cytokine genes.

Transcription Factors	*IL-8*	*IL-1β*	*CCL20*	*TNF-α*
NF-κB	nd	nd	^a^ 38–49 72–81 154–165	nd
NF-κB1	45–55	nd	62–72 71–81 197–207	191–201
AP-1	66–74	18–26 234–241	nd	nd
c-Jun	11–17 68–74 112–118 243–249	18–24 34–40 236–242	101–107 166–172	nd

The putative binding sites were predicted using Alggen promo software, V 3.0.2. The maxium dissimilarity rate between the putative and consensus sequences was set to 15%. nd: not detected. ^a^ Start and end positions of a putative conserved transcription factor binding sequences.

**Table 2:** Sequence codes for shRNAs that target human TLR10 mRNA.

Construct	5′-Antisense (19 nt)-Hairpin Loop (9 nt)-Sense (19 nt)-Termination (5 Ts)-3′
sc-40272-VA	CTCTGTGGGTGAAGAATGA*TTCAAGAGA*TCATTCTTCACCCACAGAGTTTTT
sc-40272-VB	GGAACCCATTCCATTCTAT*TTCAAGAGA*ATAGAATGGAATGGGTTCCTTTTT
sc-40272-VC	CCAGAGAAATGTATGAACT*TTCAAGAGA*AGTTCATACATTTCTCTGGTTTTT

Underlined *and italic* nucleotide sequences designate hairpin loop region of shRNAs. nt, nucleotide.

**Table 3:** Primer sequences used for qRT-PCR.

Gene	Sequence (5′~3′)
*TLR1-F*	AGTGTTTTCAAATTCAACCAGGA
*TLR1-R*	AATGACAGGTCCAAGTGCTT
*TLR2-F*	CATTCTATGCCTGAAACTTGTCA
*TLR2-R*	CCAGTGTCTTGGGAATGCAG
*TLR3-F*	GTTGACTCAGGTACCCGATGA
*TLR3-R*	ATACCTTGTGAAGTTGGCGG
*TLR4-F*	CCATTGAAGAATTCCGATTAGCA
*TLR4-R*	CAATAGTCACACTCACCAGGG
*TLR5-F*	CTTCCTGCTCCTTTGATGGC
*TLR5-R*	TCCTGATATAGTTGAAGCTCAGC
*TLR6-F*	TCATGTTCCAAAAGACCTACCG
*TLR6-R*	ACTCTGATAGAAAGCTCATGTCA
*TLR7-F*	AACTCTGCCCTGTGATGTCA
*TLR7-R*	TGAGGTTCGTGGTGTTCGT
*TLR8-F*	TCATTTCAAGGGCTGCAAAATC
*TLR8-R*	CCCGTCTGTGATATTCAAGCC
*TLR9-F*	GCTGTTCCTGAAGTCTGTGC
*TLR9-R*	ATCATGGAGGTGGTGGATGC
*TLR10-F*	TCCAGAGCTGCCAGAAGAAA
*TLR10-R*	AAATCCAGTGTCGTTGTGGC
*TNF-α-F*	TGAGCACTGAAAGCATGATCC
*TNF-α-R*	GGAGAAGAGGCTGAGGAACA
*IL-8-F*	TGCCAAGGAGTGCTAAAGAAC
*IL-8-R*	TCCACTCTCAATCACTCTCAGT
*IL-1β-F*	GGGATAACGAGGCTTATGTGC
*IL-1β-R*	AGGTGGAGAGCTTTCAGTTCA
*CCL20-F*	ACTGTGGCTTTTCTGGAATGG
*CCL20-R*	AGCACTAAACCCTCCATGATG
*β-actin-F*	GGGACCTGACTGACTACCTC
*β-actin-R*	AGCTTCTCCTTAATGTCACGC

## Supplementary Materials

Supplementary materials can be found at http://www.mdpi.com/1422-0067/17/6/859/s1.

## References

[1] Guan Y., Ranoa D.R., Jiang S., Mutha S.K., Li X., Baudry J., Tapping R.I. (2010). Human TLRs 10 and 1 share common mechanisms of innate immune sensing but not signaling. J. Immunol..

[2] Chuang T., Ulevitch R.J. (2001). Identification of hTLR10: A novel human Toll-like receptor preferentially expressed in immune cells. Biochim. Biophys. Acta.

[3] Hasan U., Chaffois C., Gaillard C., Saulnier V., Merck E., Tancredi S., Guiet C., Briere F., Vlach J., Lebecque S. (2005). Human TLR10 is a functional receptor, expressed by B cells and plasmacytoid dendritic cells, which activates gene transcription through MyD88. J. Immunol..

[4] Govindaraj R.G., Manavalan B., Lee G., Choi S. (2010). Molecular modeling-based evaluation of hTLR10 and identification of potential ligands in Toll-like receptor signaling. PLoS ONE.

[5] Lee S.M., Kok K.H., Jaume M., Cheung T.K., Yip T.F., Lai J.C., Guan Y., Webster R.G., Jin D.Y., Peiris J.S. (2014). Toll-like receptor 10 is involved in induction of innate immune responses to influenza virus infection. Proc. Natl. Acad. Sci. USA.

[6] Regan T., Nally K., Carmody R., Houston A., Shanahan F., Macsharry J., Brint E. (2013). Identification of TLR10 as a key mediator of the inflammatory response to Listeria monocytogenes in intestinal epithelial cells and macrophages. J. Immunol..

[7] Nagashima H., Iwatani S., Cruz M., Jimenez Abreu J.A., Uchida T., Mahachai V., Vilaichone R.K., Graham D.Y., Yamaoka Y. (2015). Toll-like Receptor 10 in Helicobacter pylori Infection. J. Infect. Dis..

[8] Zhang H., Tay P.N., Cao W., Li W., Lu J. (2002). Integrin-Nucleated Toll-like receptor (TLR) dimerization reveals subcellular targeting of TLRs and distinct mechanisms of TLR4 activation and signaling. FEBS Lett..

[9] Opsal M.A., Vage D.I., Hayes B., Berget I., Lien S. (2006). Genomic organization and transcript profiling of the bovine toll-like receptor gene cluster TLR6-TLR1-TLR10. Gene.

[10] Shinkai H., Muneta Y., Suzuki K., Eguchi-Ogawa T., Awata T., Uenishi H. (2006). Porcine Toll-like receptor 1, 6, and 10 genes: Complete sequencing of genomic region and expression analysis. Mol. Immunol..

[11] Bell M.P., Svingen P.A., Rahman M.K., Xiong Y., Faubion W.A. (2007). FOXP3 regulates TLR10 expression in human T regulatory cells. J. Immunol..

[12] Oosting M., Cheng S.C., Bolscher J.M., Vestering-Stenger R., Plantinga T.S., Verschueren I.C., Arts P., Garritsen A., van Eenennaam H., Sturm P. (2014). Human TLR10 is an anti-inflammatory pattern-recognition receptor. Proc. Natl. Acad. Sci. USA.

[13] Krutzik S.R., Tan B., Li H., Ochoa M.T., Liu P.T., Sharfstein S.E., Graeber T.G., Sieling P.A., Liu Y.J., Rea T.H. (2005). TLR activation triggers the rapid differentiation of monocytes into macrophages and dendritic cells. Nat. Med..

[14] Kawai T., Akira S. (2009). The roles of TLRs, RLRs and NLRs in pathogen recognition. Int. Immunol..

[15] Verma R., Jung J.H., Kim J.Y. (2014). 1,25-Dihydroxyvitamin D_3_ up-regulates TLR10 while down-regulating TLR2, 4, and 5 in human monocyte THP-1. J. Steroid Biochem. Mol. Biol..

[16] Tan R.S.T., Ho B., Leung B.P., Ding J.L. (2014). TLR cross-talk confers specificity to innate immunity. Int. Rev. Immunol..

[17] Kawai T., Akira S. (2006). TLR signaling. Cell Death Differ..

[18] Liu H., Sidiropoulos P., Song G., Pagliari L.J., Birrer M.J., Stein B., Anrather J., Pope R.M. (2000). TNF-α gene expression in macrophages: Regulation by NF-κB is independent of c-Jun or C/EBP β. J. Immunol..

[19] Kim D., Kim Y.J., Koh H.S., Jang T.Y., Park H.E., Kim J.Y. (2010). Reactive oxygen species enhance TLR10 expression in the human monocytic cell line THP-1. Int. J. Mol. Sci..

[20] Burke B., Sumner S., Maitland N., Lewis C.E. (2002). Macrophages in gene therapy: Cellular delivery vehicles and *in vivo* targets. J. Leukoc. Biol..

[21] Kusumawati A., Commes T., Liautard J.P., Widada J.S. (1999). Transfection of myelomonocytic cell lines: Cellular response to a lipid-based reagent and electroporation. Anal. Biochem..

[22] Kunkel S.L., Standiford T., Kasahara K., Strieter R.M. (1991). Interleukin-8 (IL-8): The major neutrophil chemotactic factor in the lung. Exp. Lung Res..

[23] Hieshima K., Imai T., Baba M., Shoudai K., Ishizuka K., Nakagawa T., Tsuruta J., Takeya M., Sakaki Y., Takatsuki K. (1997). A novel human CC chemokine PARC that is most homologous to macrophage-inflammatory protein-1 α/LD78 α and chemotactic for T lymphocytes, but not for monocytes. J. Immunol..

[24] Rider P., Carmi Y., Guttman O., Braiman A., Cohen I., Voronov E., White M.R., Dinarello C.A., Apte R.N. (2011). IL-1α and IL-1β recruit different myeloid cells and promote different stages of sterile inflammation. J. Immunol..

[25] Le J., Vilcek J. (1987). Tumor necrosis factor and interleukin 1: Cytokines with multiple overlapping biological activities. Lab. Investig..

[26] Farre D., Roset R., Huerta M., Adsuara J.E., Rosello L., Alba M.M., Messeguer X. (2003). Identification of patterns in biological sequences at the ALGGEN server: PROMO and MALGEN. Nucleic Acids Res..

[27] ALGGEN. http://alggen.lsi.upc.es.

