# Supplementary material for: Stable Toll-Like Receptor 10 Knockdown in THP-1 Cells Reduces TLR-Ligand-Induced Proinflammatory Cytokine Expression
Source: Int J Mol Sci. 2016 Jun 1;17(6):859. doi: 10.3390/ijms17060859 (PMC4926393; doi:10.3390/ijms17060859)
Supplement: Supplementary file 1 [file ijms-17-00859-s001.pdf]

Supplementary Materials: Stable Toll-Like Receptor 10 Knockdown in THP-1 Cells Reduces TLR-Ligand-Induced Proinflammatory Cytokine Expression

Hai Van Le and Jae Young Kim

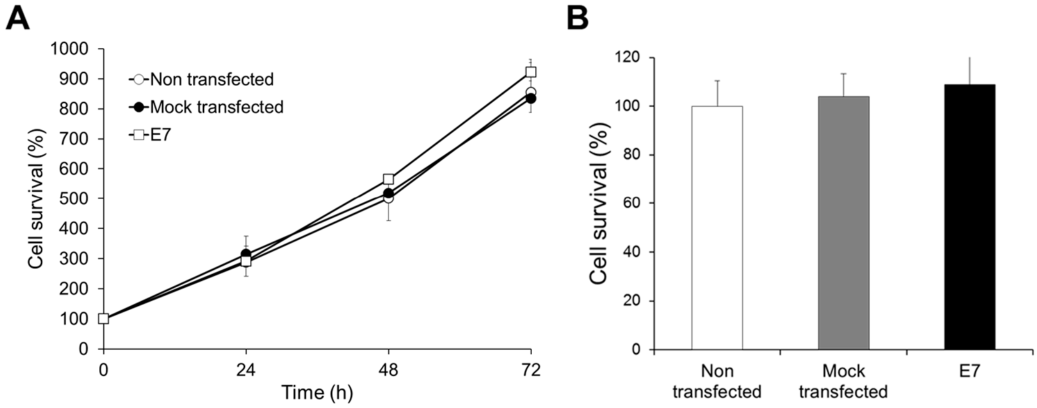

Figure S1. Growth rate of TLR10 knockdown cells. Cell viability and proliferation were determined with both trypan blue exclusion (**A**) and Ez-Cytox cell viability assay (**B**), which is based on the cleavage of the tetrazolium salt to water-soluble formazan by succinate-tetrazolium reductase. Cells were incubated with 10 μL of Ez-Cytox solution for 6 h in the 37 °C. Then, absorbance was measured using the ELISA Reader at 450 nm. There were no significant differences among three groups.

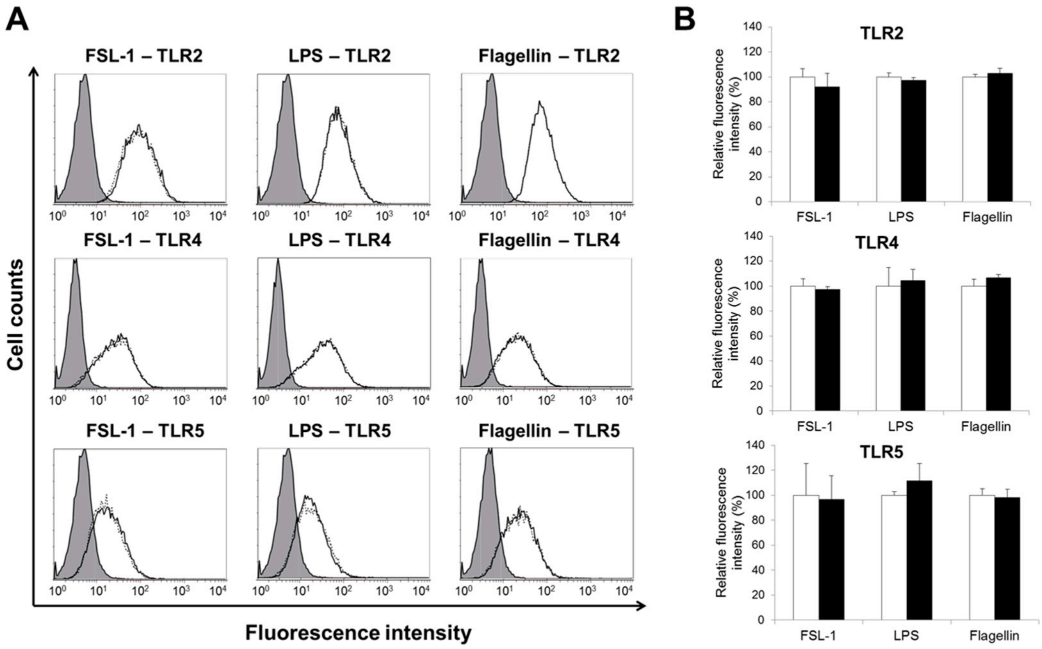

Figure S2. Cell surface expression of Toll-like receptor 2, 4, and 5 of TLR10 knockdown cells after treatment with FSL-1, LPS, and flagellin. TLR10 knockdown cells were treated with 100 ng/mL of synthetic diacylated lipoprotein (FSL-1), lipopolysaccharide (LPS) or flagellin for 4 h and were then analyzed for cell surface expression of TLR2, 4, and 5 by fluorescence-activated cell sorting (FACS). (**A**) Gray shaded area, negative control; histogram with solid line, TLR-ligand untreated cells; histogram with dotted line, TLR-ligand treated cells (**B**) White bar, TLR-ligand untreated cells; black bar, TLR-ligand treated cells.

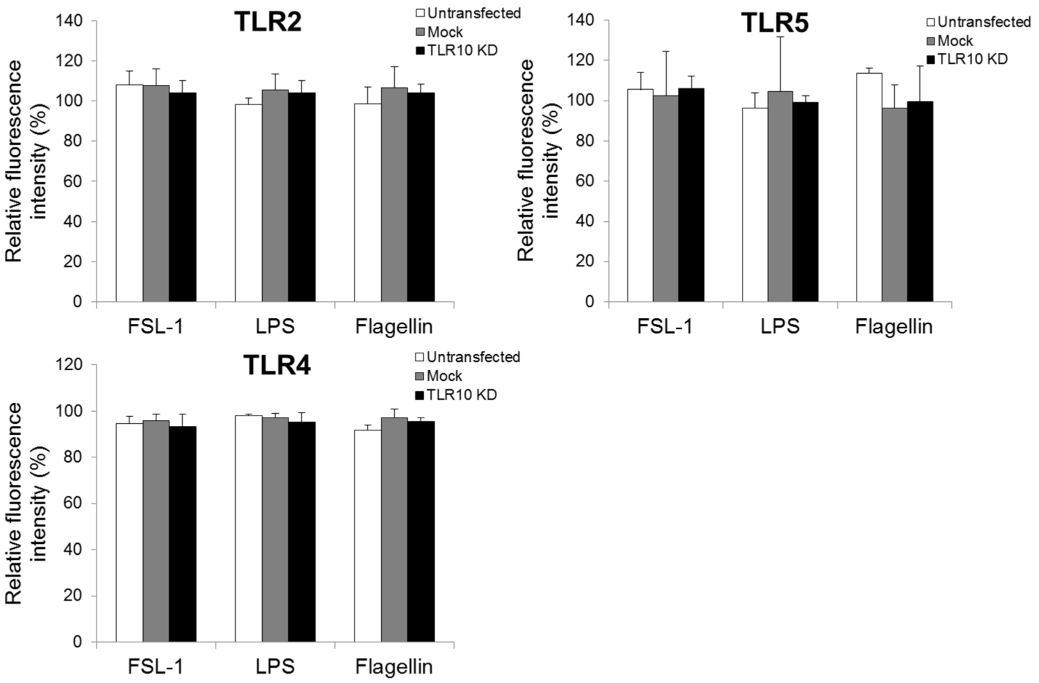

Figure S3. Cell surface expressions of TLR2, 4, and 5 of untransfected, mock-transfected and TLR10 knockdown cells after treatment with FSL-1, LPS, and flagellin. Cells were treated with 100 ng/mL of FSL-1, LPS or flagellin for 4 h and were then analyzed for cell surface expression of TLR2, 4, and 5 by FACS.
